# Tumor metabolism in pheochromocytomas: clinical and therapeutic implications

**DOI:** 10.37349/etat.2024.00222

**Published:** 2024-04-24

**Authors:** Mohammad Sadiq Jeeyavudeen, Navin Mathiyalagan, Cornelius Fernandez James, Joseph M. Pappachan

**Affiliations:** Taipei Medical University, Taiwan, China; ^1^Department of Endocrinology & Metabolism, University Hospitals of Edinburgh, EH16 4SA Edinburgh, UK; ^2^Department of Medical Oncology, Nottingham University Hospitals NHS Trust, NG5 1PB Nottingham, UK; ^3^Department of Endocrinology & Metabolism, Pilgrim Hospital, United Lincolnshire Hospitals NHS Trust, PE21 9QS Boston, UK; ^4^Department of Endocrinology and Metabolism, Lancashire Teaching Hospitals NHS Trust, PR2 9HT Preston, UK; ^5^Faculty of Science, Manchester Metropolitan University, M15 6BH Manchester, UK; ^6^Faculty of Biology, Medicine, and Health, The University of Manchester, M13 9PL Manchester, UK

**Keywords:** Pheochromocytoma, tumor metabolism, metabolomics, gene mutations, metanephrines

## Abstract

Pheochromocytomas and paragangliomas (PPGLs) have emerged as one of the most common endocrine tumors. It epitomizes fascinating crossroads of genetic, metabolic, and endocrine oncology, providing a canvas to explore the molecular intricacies of tumor biology. Predominantly rooted in the aberration of metabolic pathways, particularly the Krebs cycle and related enzymatic functionalities, PPGLs manifest an intriguing metabolic profile, highlighting elevated levels of oncometabolites like succinate and fumarate, and furthering cellular malignancy and genomic instability. This comprehensive review aims to delineate the multifaceted aspects of tumor metabolism in PPGLs, encapsulating genetic factors, oncometabolites, and potential therapeutic avenues, thereby providing a cohesive understanding of metabolic disturbances and their ramifications in tumorigenesis and disease progression. Initial investigations into PPGLs metabolomics unveiled a stark correlation between specific genetic mutations, notably in the succinate dehydrogenase complex (*SDHx*) genes, and the accumulation of oncometabolites, establishing a pivotal role in epigenetic alterations and hypoxia-inducible pathways. By scrutinizing voluminous metabolic studies and exploiting technologies, novel insights into the metabolic and genetic aspects of PPGLs are perpetually being gathered elucidating complex interactions and molecular machinations. Additionally, the exploration of therapeutic strategies targeting metabolic abnormalities has burgeoned harboring potential for innovative and efficacious treatment modalities. This review encapsulates the profound metabolic complexities of PPGLs, aiming to foster an enriched understanding and pave the way for future investigations and therapeutic innovations in managing these metabolically unique tumors.

## Introduction

Pheochromocytomas (PCCs) and paragangliomas (PGLs), together known as PCCs and PGLs (PPGLs), are catecholamine-producing neuroendocrine tumors arising from the enterochromaffin cells, embryologically originating from the neural crest of the fetus. Historically they were classified as two discrete entities by the International Agency for Research on Cancer (IARC), an agency of the World Health Organization (WHO). However, the recently published fifth WHO classification of endocrine and neuroendocrine tumors described PCCs as intra-adrenal PGLs originating from the chromaffin cells of the adrenal medulla [1]. As the pathogenesis of nearly 80% of all PGLs can have a genetic basis, genetic testing using next-generation sequencing (NGS) should be performed in all patients diagnosed with PGL [2]. Nearly 35–40% of the PGLs have germline mutations in susceptibility genes, hence achieving the highest heritability of all human tumors. Additionally, nearly 30% of the PGLs have somatic mutations [2]. Taken together, there could be more than 20 PPGLs gene mutations which can be divided into 3 main molecular clusters: pseudohypoxia cluster 1 (1A and 1B), kinase-signaling cluster 2, and wingless-type (Wnt) signaling cluster 3 [3]. These clusters have distinct biochemical, clinical, and imaging features, and varying prognosis indicating that personalized investigation and treatment is a possibility [3].

The distinct characteristics in the cellular metabolic pathways of PPGLs have important connotations in their pathobiological behavior, metastatic potential, clinical presentation, and therapeutic response. Several of the aforementioned genetic abnormalities also directly or indirectly contribute to structural and/or functional alterations in the cellular enzyme machinery which results in abnormal metabolic products and finally tumorigenesis. This review explores the metabolic pathways, their interlink to the genetic defects, and the related alterations in tumor behavior in PPGLs, with special emphasis on molecular, clinical, and therapeutic implications.

## PPGLs: tumor metabolomics

Metabolomics is the science dealing with the characteristics of the cell’s metabolic pathways, their genetic features, transcriptional functions, and protein byproducts [4]. It is important to understand these features for predicting the clinical, biochemical, and biological aspects of tumors especially neuroendocrine tumors which possess specific metabolic characteristics. Pollard et al. [5], through proton nuclear magnetic resonance (^1^H-NMR) spectroscopy, illustrated crucial findings such as the elevation of succinate in SDHB-related PGLs. Understanding these unique metabolic characteristics illustrates the pathophysiological processes inherent in PPGLs and provides a framework for exploring targeted therapeutic strategies and diagnostic markers [6]. D-2-hydroxyglutarate (D-2HG) oncometabolite is generated by the neomorphic activity of the mutated isocitrate dehydrogenase (IDH) [7]. Correlations, such as its association with a distinct cytosine-phosphate-guanine (CpG) island methylator phenotype (CIMP) and its regulatory impacts on α-ketoglutarate-dependent dioxygenases, spotlight its multifaceted role within oncogenic processes [8]. Analysing the downstream effects and biological interactions of D-2HG provides a platform for exploring oncogenic metabolites and their subsequent influences on tumorigenesis and tumor progression [7].

Technological advancements, specifically in the realm of mass spectrometry (MS), have facilitated a deeper understanding of the metabolomic study of biological samples. Transitioning from gas chromatography to liquid chromatography (LC) has enabled the analysis of biologically active polar molecules paving the way for a more nuanced understanding of metabolites and their roles within biological systems [9]. Especially within the context of PPGLs, methodologies like LC-tandem MS (LC-MS/MS) provide an invaluable tool for investigating tumor metabolites, guiding research toward more targeted diagnostic and therapeutic applications [10]. The largest metabolomic study to date was done by Richter et al, analyzing 395 fresh frozen PPGL samples and measuring various metabolites, including citrate, isocitrate, cis-aconitate, α-ketoglutarate, the D-2HG, the L-2-hydroxyglutarate (L-2HG), succinate, fumarate, malate, pyruvate, lactate, glutamate, glutamine, aspartate, and asparagine [11]. The clustering analysis discerned two primary clusters: the inaugural cluster, characterized by heightened succinate and predominantly involving succinate dehydrogenase complex (*SDHx*)-related tumors, and a second cluster which is further subdivided into two sub-clusters, each differentiated primarily by their respective concentrations of citrate, isocitrate, and cis-aconitate. The first sub-cluster comprising tumors linked to hypoxic signaling including von Hippel-Lindau (*VHL*)*,* endothelial Per-Arnt-Sim (PAS) domain protein 1 (*EPAS1*)*,* fumarate hydratase (*FH*)*,* and *IDH*-related tumors presented diminished levels of citrate, isocitrate, and cis-aconitate. Conversely, the second sub-cluster, mainly involving tumors related to kinase signaling such as those related to rearranged during transfection (*RET*)*,* neurofibromatosis type 1 (*NF1*)*,* and transmembrane protein 127 (*TMEM127*), displayed elevated concentrations of the aforementioned metabolites [11].

Thus, the use of both LC-MS/MS and immunohistochemistry plays a pivotal role in deciphering the pathogenicity of gene variants, especially within the context of uncertain significance identified by multipanel or exome sequencing. Harmonizing metabolic profiles with immunohistochemistry results provides a layered understanding, offering insights into the functional impacts of certain gene variants. This dual-faceted approach not only aids in discerning the pathogenicity of variants but also offers a detailed view of the molecular underpinnings and potential biomarkers related to PPGLs.

The metabolic pathways involved in hormone synthesis and processing within the adrenal medulla and sympathetic ganglia are complex and finely regulated to ensure a proper physiological response to stressors [12]. The synthesis of catecholamines begins with the amino acid tyrosine, which enters the adrenal medulla and sympathetic neurons through the L-type amino acid transporter (LAT) 1 and LAT2 [13]. Through a series of enzymatic reactions, tyrosine is converted to dihydroxyphenylalanine (DOPA) by the enzyme TH, which is the rate-limiting step in catecholamine synthesis [14]. DOPA is then converted to dopamine by DOPA decarboxylase (DDC). In the cytoplasm of adrenal medullary cells and sympathetic neurons, dopamine is transported into storage vesicles, where it is further hydroxylated to norepinephrine by dopamine β-hydroxylase (DBH) [13]. In the adrenal medulla, norepinephrine can be methylated to form epinephrine through the action of phenylethanolamine N-methyltransferase (PNMT) [15]. Once synthesized, catecholamines are stored in vesicles until they are released into the bloodstream in response to various stimuli. A sizeable fraction does leak into the cytoplasm where it is metabolized into metanephrines. The release process is triggered by the arrival of a nerve impulse which causes the vesicles to fuse with the cell membrane and release their contents into the bloodstream. Most epinephrine (around 90%) of the body’s requirement comes from the adrenal medulla and norepinephrine comes from the sympathetic ganglia. Less than 10% of the body’s norepinephrine comes from the adrenal medulla [15].

**Figure 1:**
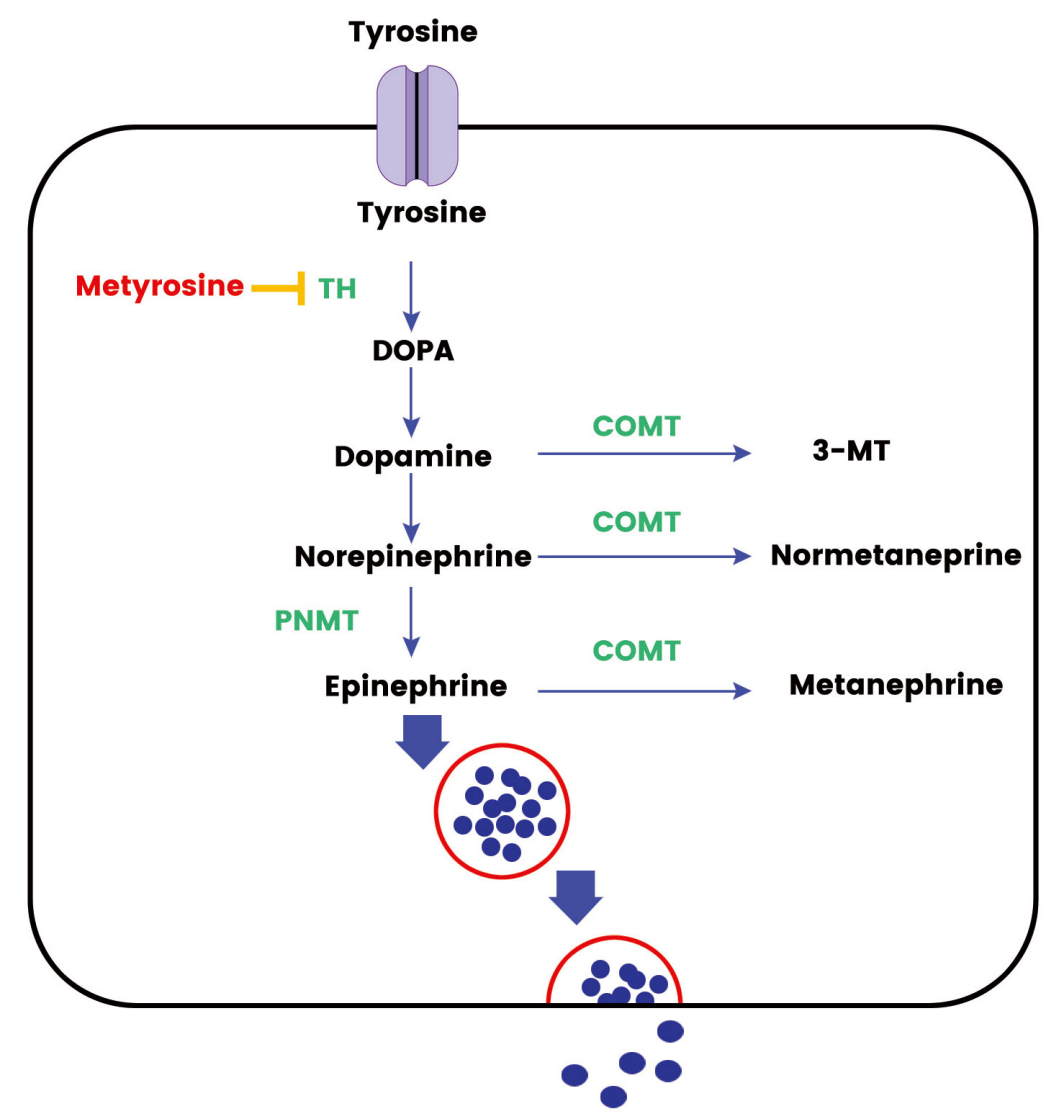
The synthesis, release, and metabolism of adrenal medullary hormones. TH: tyrosine hydroxylase; COMT: catechol-*O*-methyltransferase; 3-MT: 3-methoxytyramine. Metyrosine inhibits the action of TH and Cortisol activates the PNMT activity in adrenal medulla

The synthesis and secretion of catecholamines are tightly regulated by various factors. The activity of TH, for instance, is modulated by the level of catecholamines through a negative feedback mechanism, ensuring the balance in catecholamine levels is maintained. Additionally, other hormonal and neural mechanisms also play a significant role in the regulation of catecholamine synthesis and secretion. In basal conditions the catecholamines function as metabolic hormones and upon stressful stimulus, they exhibit a fight and flight response triggering the cascade of events for release from the stored vesicle [12]. The main rate-limiting enzyme is the TH. *TH* gene, harboring 13 exons, is situated at 11p15.5 on chromosome 11 and gives rise to four isoforms through alternative messenger RNA (mRNA) splicing [14]. Through its pivotal role in catecholamine synthesis and multifaceted regulation, *TH* has attracted significant attention across various cancer research areas. Recent investigations have revealed multiple *TH* polymorphisms in the general population are associated with heightened norepinephrine levels and elevated blood pressure [16]. TH, with an approximate molecular weight of 240 kDa, is largely relegated to adrenal, extra-adrenal chromaffin cells, and postganglionic sympathetic nerve endings. TH relies on cofactors like ferric (Fe^3+^), tetrahydrobiopterin, and molecular oxygen for the hydroxylation step. Originating from guanosine triphosphate (GTP), tetrahydrobiopterin donates hydrogen atoms to keep TH reduced and active [14]. The Figure 1 shows the graphical representation of the synthesis, release, and metabolism of adrenal medullary hormones.

The complexity of controlling catecholamine synthesis involves a nuanced regulation of TH activity, combining short-term post-transcriptional mechanisms with long-term transcriptional ones. The former encompasses enzyme phosphorylation, dephosphorylation, feedback inhibition by catecholamines, and ubiquitination, while the latter primarily engages transcriptional mechanisms [16]. TH degradation is postulated to be mediated by the ubiquitin–proteasome pathway [14]. Catecholamines mitigate TH formation in its reduced, active state through the oxidation of tetrahydrobiopterin to pteridine and also serve as antagonists of tetrahydrobiopterin, obstructing the TH catalytic domain [17]. Short-term TH activity regulation is further realized via the phosphorylation and dephosphorylation of up to four serine residues at its regulatory site, with phosphorylation facilitated by numerous kinases [like protein kinase A (PKA), PKC, Ca^2+^/calmodulin-dependent protein kinase II (CaMKII), mitogen-activated protein kinase-activated protein kinase 2 (MAPKAP-K2), extracellular signal-regulated kinase 1 (ERK1), ERK2, mitogen- and stress-activated kinase 1 (MSK1), p38-regulated/activated protein kinase (PRAK)] freeing it from the catecholamine-induced feedback inhibition and thereby spurring enzyme activity [17].

Moreover, protein phosphatase 2A (PP2A) and, to a lesser degree, PP2C facilitate dephosphorylation, reinstating catecholaminergic suppression of TH [18]. This inhibition is mediated through alpha2-adrenergic or D2-dopaminergic receptors, activating cyclic adenosine monophosphate (cAMP) or Ca^2+^/calmodulin-dependent protein phosphatases [17]. Persistent stimulation of catecholamine synthesis catalyzes TH protein synthesis induction via several cAMP-dependent pathways that trigger *TH* gene transcription. An overview of the regulation of synthesis and secretion of adrenal medullary hormones is given in Table 1.

**Table 1:** Regulation of synthesis and secretion of adrenal medullary hormones

Feature	Adrenal medulla	Sympathetic ganglia
Primary hormones produced	Epinephrine and norepinephrine	Norepinephrine
Enzymes involved	TH	TH
	DDC	DDC
	DBH	DBH
	PNMT	PNMT is not significantly expressed
Key regulatory genes	*TH* gene	*TH* gene
	*DDC* gene	*DDC* gene
	*DBH* gene	*DBH* gene
	*PNMT* gene	Not significant
Regulatory mechanisms	Negative feedback by catecholamines	Negative feedback by catecholamines
	Neural control	Neural control
	Hormonal control (e.g., glucocorticoids)	Hormonal influence might be less pronounced

In adrenal medullary chromaffin cell vesicles, the norepinephrine undergoes conversion to epinephrine via the enzymatic action of PNMT [15]. PNMT utilizes S-adenosylmethionine as a co-substrate and methyl donor. Not exclusive to a single substrate, PNMT also participates in the production of additional N-methylated trace amines [19]. The regulatory control of PNMT expression is orchestrated through mechanisms mediated by the glucocorticoid receptor, in synergy with an array of transcription factors like early growth response-1 (Egr-1), activator protein 2 (AP2), specificity protein 1 (Sp1), and Myc-associated zinc-finger protein (MAZ) [20]. Ensuring elevated circulating glucocorticoid levels, the closeness of adrenocortical cells to the adrenal medulla allows glucocorticoids to diffuse passively through the chromaffin cell membrane [20]. Upon binding to the intracellular glucocorticoid receptor, the hormone-receptor complex transitions into the cell nucleus, associating with the glucocorticoid response element of the promoter region of the *PNMT* gene, situated at chromosome 17q12, thereby initiating gene transcription [21]. This mechanism elucidates why the adrenal gland is paramount as the principal source of epinephrine in the body, while extra-adrenal PNMT expression remains confined to a select group of neurons in the clinical nurse specialist (CNS) and a subset of cardiomyocytes.

Kimura et al. [22] demonstrated that while *TH* mRNA was identifiable in all patients with PCC samples, the presence of *PNMT* mRNA was exclusively observed in epinephrine-type tumors. Immunohistochemical analysis involving 70 PPGLs-wherein every PCC possessed cells showcasing immunoreactivity to *TH*, with *PNMT* expression being isolated to epinephrine-producing PCC type alone [22]. Hence PNMT still retains its capacity not only in physiological conditions but in tumor states when the production of hormone from the lesion is increased by multiple folds. In a study involving array comparative genomic hybridization (CGH) and gene expression profiling of 12 fresh frozen PPGLs samples, it was observed that while most cases had limited copy number aberrations irrespective of their malignancy status, 390 genes were differentially expressed between benign and malignant tumors [23]. Notably, the expression of *PNMT*, was significantly reduced in malignant PPGLs. This key finding positions *PNMT* downregulation as a potential hallmark of malignancy in PPGLs and underscores its prominence as a differentially expressed gene between the two tumor types [23].

Metyrosine, a recognized TH blocker, has been validated for its efficacy and safety, particularly in pre-surgical or pre-intervention contexts [24]. Metyrosine not only demonstrates a propensity to enhance intraoperative hemodynamics but also substantively mitigates catecholamine-mediated symptoms in a subset of patients [25, 26]. The strategic integration of metyrosine into treatment regimens may be particularly beneficial for specific patient cohorts. These cohorts include individuals who persistently exhibit hypertension despite the initiation of alpha-adrenergic blockade; those who exhibit intolerance towards alpha-adrenergic blockade; patients are identified as high-risk due to tumor size or location, which predisposes them to substantial catecholamine release; and patients scheduled for interventions such as chemotherapy or ablative therapy, where a significant catecholamine release is plausible [27]. Consequently, metyrosine provides a robust option to safeguard against potential clinical complications associated with catecholamine surges in these patient profiles. Hence a thorough knowledge of the metabolism of the PPGL can be used to prognosticate patients in difficult circumstances and offer additional therapeutic advantages.

The adrenal medulla generates and releases not only catecholamines but also a wide array of enzymes, peptides, and proteins that oversee catecholamine synthesis and secretion through autocrine and paracrine actions, some of which are neuropeptide Y (NPY), adrenomedullin (AM), peptides pituitary adenylate cyclase-activating polypeptide (PACAP), and catestatin [20]. NPY, a 36-amino acid neuropeptide distributed broadly within the brain and sympathetic nervous system, stimulates catecholamine secretion by enhancing *TH* expression and intracellular calcium levels [28]. AM, a peptide initially isolated from a PCC, plays a crucial role in both autocrine and paracrine activities by binding to various receptors, subsequently augmenting the adrenal blood flow and stimulating catecholamine release, along with other systemic effects such as vasodilatation and natriuresis stimulation [29–31]. The concentration of AM is also markedly increased in patients with adrenal PCC and can be a usual biomarker differentiating between PPGL in certain special patients when there is an imaging constraint [31]. PACAP, another neuropeptide, amplifies catecholamine secretion through transcription induction and the activation of biosynthetic enzymes like TH, DBH, and PNMT, also influencing the expression and secretion of additional peptides like brain natriuretic peptide and enkephalins in normal adrenal medullary chromaffin cells [32, 33]. It is very important to note that PNMT expression is mainly influenced by genetic backgrounds rather than tumor location [34]. Lastly, catestatin, derived from the proteolytic cleavage of chromogranin A, predominantly acts as a noncompetitive nicotinic cholinergic antagonist, thereby providing robust negative feedback inhibition of catecholamine secretion [33]. These peptides collectively modulate chromaffin cell functionality through an array of membrane receptors, primarily belonging to the G protein-coupled receptors (GPCRs) family, orchestrating a complex network of hormonal regulation and release within the adrenal medulla [35].

## Molecular defects and metabolic byproducts of genetic mutations in PPGLs

Reports indicate that an annual PPGL incidence ranges from two to eight cases per million people, while in hypertensive patients, PPGL prevalence is estimated to be between 0.2% and 0.6% [36]. Notably, 5% of patients with adrenal masses incidentally identified through radiology were found to have PPGLs [36]. Molecular analysis demonstrates that there is an elevated gene expression tied to cellular cycle, tumor evolution, metastasis, hypoxia, angiogenesis, and the Wnt signaling pathway in PPGLs when contrasted with normal adrenal glands [10, 12, 37, 38]. Around 35–40% of PPGLs exhibit a hereditary predisposition due to germline mutations in more than a dozen susceptibility genes [39]. Additionally, large genomic studies reveal approximately 30% of cases possessing tumor-specific somatic mutations, thus identifying tumorigenesis drivers in roughly 60–70% of cases [40, 41]. Identifying pathogenic variants in susceptibility genes is pivotal as it modifies the patients’ and potentially asymptomatic relatives’ required surveillance, screening, and clinical care [42]. These susceptibility genes fall into three main clusters: pseudohypoxia cluster (cluster 1), Kinase signaling cluster (cluster 2), and Wnt signaling cluster (cluster 3) [38, 43–45].

### Pseudohypoxia cluster (cluster 1)

The pseudohypoxia cluster incorporates genes pivotal for cellular hypoxia response, such as *VHL, SDHx (SDHA, SDHB, SDHC, SDHD), FH,* malate dehydrogenase 2 (*MDH2*)*,* Egl nine homolog 1 (*EGLN1*)*,* and *EGLN2* [38]. Normally, these genes synergize to maintain the oxygen levels and hypoxia-inducible factors (HIFs) activity within the cell. HIFs, acting as transcription factors, manipulate gene expression, orchestrating cellular metabolism, angiogenesis, and cell survival [46]. In the context of PPGLs, mutations within the pseudohypoxia cluster can instigate HIF accumulation, potentially propelling angiogenesis and tumor expansion [46, 47]. Intriguingly, mutations in *SDHx* genes culminate in succinate or fumarate accumulation, inhibiting prolyl hydroxylases and stabilizing HIFs and mimics a hypoxic state within cells, despite the presence of normal oxygen levels—a phenomenon termed pseudo-hypoxia [5, 48, 49]. This altered cellular environment drastically influences various metabolic pathways, significantly impacting the catecholamine biosynthesis pathway, which is integral to the normal function of the adrenal medulla and sympathetic ganglia [50].

Moreover, the tumor’s metabolic shift towards glycolysis, driven by HIF stabilization, is akin to the Warburg effect observed in many cancer cells [46]. This altered metabolic state could lead to cachexia or other metabolic syndromes, further complicating the clinical presentation [2, 42]. Besides, the hypoxia-driven angiogenesis promotes tumor growth and potentially facilitates metastatic spread, which if occurs, could present with symptoms related to organ involvement [47]. Cluster 1 disease is further delineated into subcategories, cluster 1A and cluster 1B, predicated upon differentially expressed genes [12]. PPGLs exhibiting mutations in *SDHx* and *VHL* are respectively sub-categorized therein.

### Kinase signaling cluster (cluster 2)

The kinase signaling cluster, encompassing genes such as *NF1, RET,* MYC associated factor X (*MAX*)*,* and *TMEM127*, plays a vital role in controlling cell growth and proliferation under typical conditions [47]. The Kinase-Signaling cluster, fundamental in manipulating cellular signaling pathways like rat sarcoma virus (RAS)/rapidly accelerated fibrosarcoma (RAF)/mitogen-activated extracellular signal-regulated kinase (MEK)/ERK and mechanistic target of rapamycin (mTOR), experiences disruptions due to genetic defects, upsetting cellular proliferation, differentiation, and apoptosis equilibriums [51, 52]. The *NF1* mutations can dysregulate the RAS signaling pathway, inducing rampant cellular proliferation, a trademark of tumorigenesis [53]. Likewise, *RET, TMEM127,* and *MAX* gene mutations have been implicated in perturbed cellular signaling, highlighting the kinase-signaling cluster’s instrumental role in comprehending PPGL tumor characteristics [38, 42, 54]. Similar to cluster 1 disease, cluster 2 disease has been further divided into subclusters: cluster 2A (presenting mutations in *RET, NF1,* and *TMEM127*), cluster 2B (sporadic tumors), and cluster 2C (displaying mutations in *VHL* 3.7% and *RET* 11.1%, plus sporadic tumors) [55, 56].

### Wnt signaling cluster (cluster 3)

**Figure 2:**
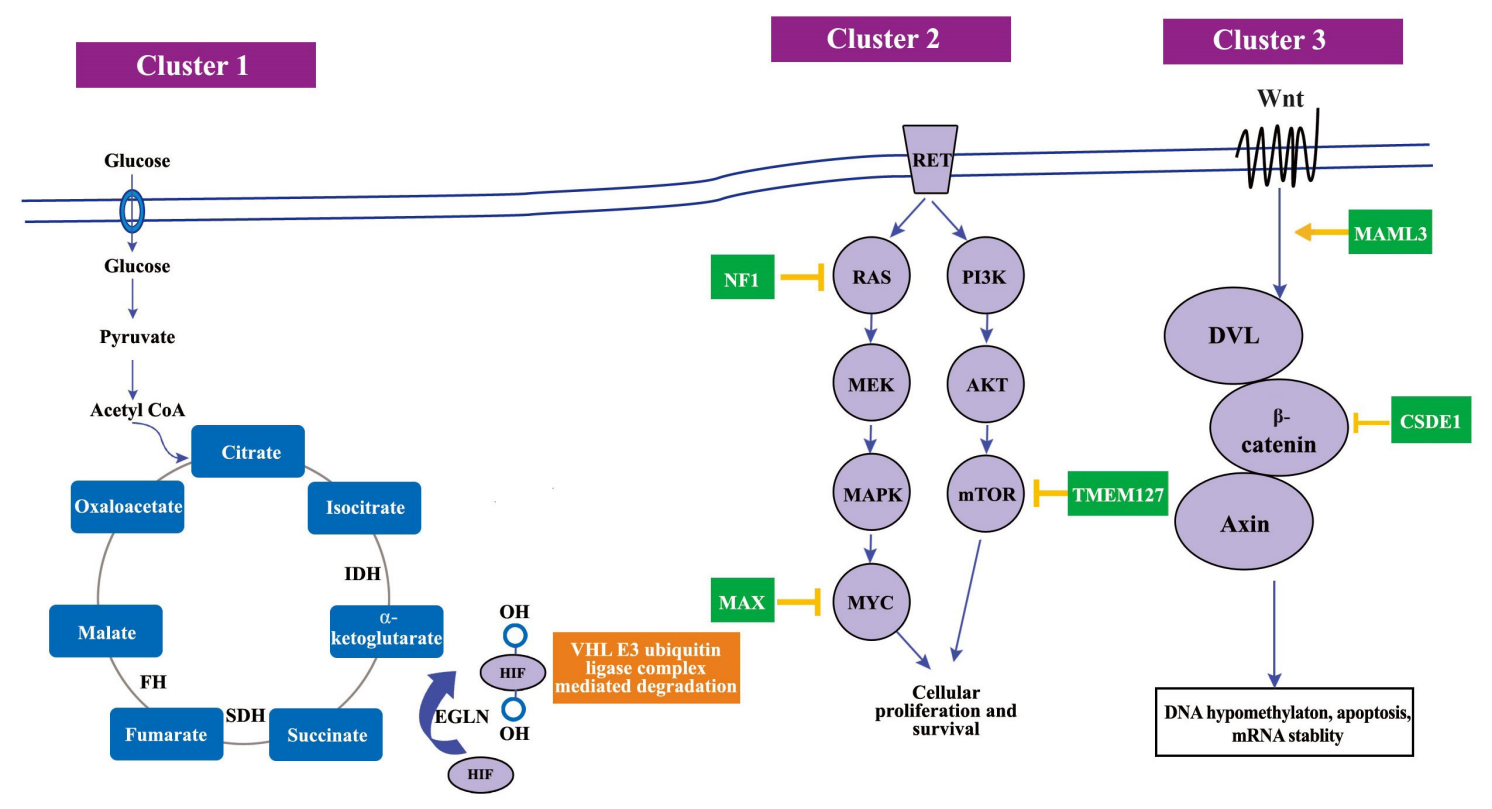
The molecular pathways in the PPGL pathogenesis. SDH: succinate dehydrogenase; PI3K: phosphoinositide 3-kinase; AKT: protein kinase B; MAPK: mitogen-activated protein kinase; MYC: myelocytomatosis oncogene; Wnt: wingless-related integration site; DVL: Dishevelled. Mutations in cluster 1 genes lead to an accumulation of oncometabolites, which results in decreased HIF-α degradation, mimicking a state of hypoxia and therefore referred to as a pseudohypoxic state. Cluster 2 gene mutation affects cellular proliferation defect and cluster 3 gene mutations affects DNA hypomethylation and mRNA stability

The Wnt Signalling cluster, distinguished by mutations in cold shock domain-containing protein E1 (*CSDE1*) and somatic gene fusions of mastermind-like transcriptional coactivator 3 (*MAML3*), is crucial in shaping PPGLs’ tumor features [47]. Governed by these genetic modifications, the Wnt/β-catenin signaling pathway is imperative for cellular differentiation and proliferation [52]. Discrepancies in this pathway owing to genetic defects can foster anomalous cellular differentiation, thereby aiding the tumorigenic process [57]. Discerning the influence of the Wnt signaling cluster on PPGLs’ tumor characteristics is vital for sculpting innovative diagnostic and therapeutic approaches. Investigating the relationship between genetic mutations within this cluster, the Wnt/β-catenin signaling pathway, and its interaction with other signaling streams, unveils a convoluted molecular web that governs tumor behavior in PPGLs [24, 38, 43, 47]. A thorough grasp of these molecular interplays is crucial for programming PPGLs’ clinical management. In Figure 2, it shows the various gene clusters and various the molecular pathways in the pathogenesis of PPGL.

An overview of the various genes implicated in the PPGL pathogenesis, their impact on the tumorigenesis and the functional imaging specific to the cluster types is given in Table 2.

**Table 2:** Various genes implicated in the PPGL pathogenesis, their impact on the tumorigenesis and the functional imaging specific to the cluster types

**Cluster type**	**Sub-type**	**Germline or somatic**	**Exclusively somatic**	**Location of gene**	**Normal function**	**Impact of mutations**	**Nuclear imaging**
Pseudohypoxia	Cluster 1A	*SDHA* *SDHB* *SDHC* *SDHD* *SDHAF2* *FH* *MDH2* *IDH3B* *GOT2* *DLST* *SLC25A11* *SUCLG2*	*IDH1* *IDH2*	5p15 1p36.1-p35 1q23.3 11q23.1 11q12.2 1q42.1 7q11.23 20p13 16q21 14q24.3 17p13.2 3p14.1 2q34 15q26.1	Regulate oxygen levels and HIF activity	HIF accumulation, propelling angiogenesis, and tumor expansion	^68^Ga-DOTATATE positron emission tomography/computed tomography (PET/CT)
	Cluster 1B	*VHL* *EGLN1* *EGLN2*	*EPAS1*	3p25.3 1q42.2 19q13.2 2p21	Regulate oxygen levels and HIF activity	HIF accumulation, propelling angiogenesis, and tumor expansion	^18^F-FDOPA imaging
Kinase signaling	Cluster 2A and 2B	*NF1* *RET* *MAX* *TMEM127* *MET* *MERTK*	*FGFR1* *HRAS* *BRAF*	17q11.2 10q11.21 14q23.3 2q11.2 7q31 2q14.1 8p11.23 11p15.5 7q34	Control cell growth and proliferation	Cell proliferation, differentiation, and apoptosis	^18^F-FDOPA imaging
Wnt signaling	None	None	*CSDE1* *UBTF-MAML3*	1p13.3 4q31.3	Regulate cellular differentiation and proliferation	Anomalous cell differentiation and tumorigenesis	None

GOT2: glutamic-oxaloacetic transaminase 2; DLST: dihydrolipoamide succinyltransferase; SLC25A11: solute carrier family 25 member 11; SUCLG2: succinate-CoA ligase GDP-forming subunit beta 2; MET: mesenchymal-epithelial transition factor; MERTK: myeloid-epithelial-reproductive tyrosine kinase; FGFR1: fibroblast growth factor receptor 1; HRAS: Harvey rat sarcoma viral oncogene homolog; BRAF: B-type Raf kinase; UBTF: upstream binding transcription factor; ^68^Ga-DOTA-TATE: ^68^Gallium-DOTA-Tyr3-octreotate; ^18^F-FDOPA: 6-L-^18^F-fluorodihydroxyphenylalanine

## Clinical presentation of PPGLs

The clinical presentation for the inherited group substantially varies from the sporadic group in the realm of PPGLs. Notably, age stands out as an important factor, with those harboring hereditary PPGLs typically developing the disease roughly a decade earlier than their sporadic counterparts [58]. However, there is a significant variation in the age at initial diagnosis among patients with hereditary syndrome. The hereditary cluster 1 PPGL patients has a very early age at first diagnosis (28.7 years) than hereditary cluster 2 (46.8 years) and sporadic PPGL patients (52.9 years) with the age at initial diagnosis of cluster 2 PPGL being almost like that of sporadic patients [59]. Multiple PGLs are much more common in the hereditary group than the sporadic PPGLs [2, 57]. A considerable 17% to 85% of hereditary PPGL patients manifest with multiple PGLs with only 1.2% prevalence in sporadic occurrences [1, 2, 55, 60].

Furthermore, *VHL*-related PGLs secrete only norepinephrine, providing a unique biochemical signature to this hereditary variant of the disease [45]. Gender disparities in occurrence also take the forefront in distinguishing between hereditary and sporadic forms. Hereditary PGLs are common in both genders, while sporadic instances have a female predominance, with a notable 71% to 29% female-to-male ratio [60]. Bilateral PCCs, which are characteristic of *RET, VHL,* and *NF1* genes, do not co-exist with multiple PGLs, a phenomenon commonly associated with *SDHx* syndromes [61]. Hence, patients presenting with multiple PGLs are almost certain to be diagnosed with a hereditary form of the disease. Moreover, among patients with sympathetic PGLs, a substantial 25% have the presence of a hereditary syndrome (Table 3) [62].

**Table 3:** Various hereditary syndromes associated with PPGL

Syndromes	Gene involved	Clinical features
VHL syndrome	*VHL*	Hemangioblastomas of the CNS, clear cell renal cell carcinoma, endolymphatic sac tumor, pancreatic tumors, epididymal cystadenomas, broad ligament cystadenoma, and PCCs
Multiple endocrine neoplasia type 2 (MEN2)	*RET*	Medullary thyroid carcinoma, PCCs, parathyroid adenomas/hyperplasia
NF1	*NF1*	Neurofibromas, optic pathway gliomas, pigmented skin lesions (cafe-au-lait spots, freckling), Lisch nodules, PCCs, and other malignancies
Hereditary PGL/PCC syndrome (HPPS) or familial PGL (FPGL) syndrome	*SDHD*-FPGL1; *SDHAF2*-FPGL2; *SDHC*-FPGL3; *SDHB*-FPGL4; *SDHA*-FPGL5	Head and neck PGLs (HNPGLs), thoracic/abdominal/pelvic PGLs, PCCs, renal cell carcinoma, and gastrointestinal stromal tumors
Carney’s triad	c-kit (*CD117*) mutations	Gastrointestinal stromal tumors, pulmonary chondromas, PGLs
Pacak-Zhuang syndrome	*EPAS1*	PGLs, somatostatinomas, polycythemia
Hereditary leiomyomatosis and renal cell cancer (HLRCC) syndrome	*FH*	Cutaneous and uterine leiomyomas, renal cell carcinoma, uterine leiomyosarcoma

### Significance of the genetic basis of PPGL in clinical practice

From a clinical perspective, alterations in PPGL genes can be classified into two groups:

(1). The group (*RET, VHL, NF1, SDHD, SDHAF2, SDHC, SDHB, SDHA, TMEM127, MAX*) with well-defined correlations between genotype and phenotype [45, 58, 63].
(2). Constantly evolving genes that lack significant genotype-phenotype association [45, 58, 63].

PPGLs have a robust genetic inheritance, which emphasises the need for germline genetic testing [45, 58]. Genetic studies assessment for alterations in the 10 relevant genes has an impact on the selection of imaging, biochemical tests, and therapy decisions [45, 52, 58, 64]. Magnetic resonance imaging (MRI) scan from the base of the skull to the pelvis is suggested if any pathogenic changes are found in the *SDHA, SDHB,* or *SDHD* genes. It is advisable to do an MRI scan from the base of the skull to the neck when pathogenic mutations of *SDHC* and *SDHAF2* are identified [45]. *MAX* and *TMEM127* pathogenic mutation detection necessitate abdominal MRI [45]. For pathogenic gene variants in other groups of PPGL that lack established genotype-phenotype correlation general medical testing, surveillance, and family screening are recommended [45].

### Personalised patient care based on genetic profile

Customising therapy according to an individual’s genetic profile is crucial due to the expanding knowledge of novel genes and pathomechanisms, which have a role in determining anatomical location, biochemical features, metastatic risk, and prognosis [45, 65]. Individuals with germline *SDHB* mutations have an increased susceptibility to malignant tumors due to their genetic makeup [45, 65]. *FH* [66], *MAX* [67], *SDHD* [68], *SLC25A11* [69], and telomerase reverse transcriptase (*TERT*) [70, 71] mutations as well as *MAML3* gene fusion [72] have been identified in malignant PPGLs. An additional risk factor for the clinical development of PPGL is the aggressive phenotype that is linked to somatic mutations of alpha-thalassemia/mental retardation syndrome X-linked (*ATRX*) [73, 74].

CT and MRI are used for the initial localization of tumors [45]. Understanding the pathological origins of different types of PPGLs can significantly influence diagnostic imaging outcomes. The application of PET scanning in conjunction with radionuclide-based nuclear imaging is favoured for the preliminary assessment of *SDHx* mutation carriers, localization of tumors, and staging [45]. The anatomical site, genetics, and metabolic properties of PPGL all influence the choice of nuclear imaging modality and PET tracer [3, 75, 76].

Given the high level of somatostatin receptor 2 (SSTR2) expression in cluster 1A of *SDHx* mutation-related PPGL, SSTR analogue ^68^Ga-DOTATATE PET/CT provides the most sensitive functional imaging tool for the detection and analysis [3, 75]. The sensitivity of 18fluorine-fluorodeoxyglucose (^18^F-FDG)-PET imaging to detect PPGL associated with *SDHx* is notable, particularly in rapidly advancing or metastatic disease. ^18^F-FDOPA-PET is a prospective diagnostic tool for *SDHD*-associated HNPGLs owing to the exclusive absorption of ^18^F-FDOPA by parasympathetic HNPGLs [3, 77–80]. Additionally, ^18^F-FDOPA-PET is sensitive in identifying PPGL linked to the *FH* mutation.

PPGLs related to cluster 1B including *VHL, EPAS1,* and prolyl hydroxylase domain 1 and 2 genes (*PHD1/2*) show stronger L-type amino-acid transporter than SSTR2, making ^18^F-FDOPA imaging more sensitive for these tumors [77, 81, 82]. Patients with cluster 2 PPGL including MEN2, or NF1 syndrome should receive ^18^F-FDOPA-PET or ^123^I-MIBG imaging [83]. The ^123^I-MIBG scan is a required test for patients being considered for ^131^I-metaiodobenzylguanidine (^131^I-MIBG) treatment. It should be noted that MIBG scintigraphy has lower sensitivity compared to ^68^Ga-DOTATATE-PET and ^18^F-FDG-PET in diagnosing *VHL* and *SDHx* mutation-related PPGL as well as in detecting metastatic lesions. Additionally, predicting MIBG uptake solely based on the genetic background of PPGL is challenging [84–87].

## Biochemical evaluation

Due to its precision and consistency, LC-MS/MS has emerged as the benchmark. A value three times greater than the upper limit of normal values signifies a positive outcome [52]. Nonetheless, delayed diagnosis may result from the presence of pseudo-silent PPGL and a heavy tumor burden in certain patients. Hence, any findings beyond the standard range should be regarded as indicative of PPGL especially when prospectively screening for hereditary disease. Biochemical profiling is useful for assessing PPGL syndromes, as tumors can be classified based on their profile (Table 4) [52].

**Table 4:** Classification of PPGL based on biochemical and clinical profile

Biochemical type	Associated PPGL	Lab characteristics	Clinical points
Truly biochemically silent phenotype	Mostly seen in *SDHx* syndromes	No rise in metanephrines	Mostly associated with head and neck tumors
Biochemically pseudo-silent phenotype	Usually happen with very small (less than 5–7 mm) PPGLs	Levels of metanephrines can be normal or near normal, in a misleading way	None
Noradrenergic phenotype	Commonly seen in the cluster 1/pseudohypoxia group, including both *VHL* and *SDHx* mutations	Elevated normetanephrines	Sustained hypertension and tachycardia are the most common symptoms. Commonly located outside the adrenals
Adrenergic phenotype	Commonly seen in the cluster 2/kinase signaling group	Elevated metanephrine or mix of metanephrine/normetanephrine. The latter phenotype can be identified when the plasma free metanephrines are greater than 10% of the sum of metanephrine and normetanephrine	Adrenergic PPGLs are often located in the adrenal gland
Dopaminergic phenotype	Commonly seen in *SDHx* mutations, especially in *SDHB*	High levels of 3-MT with normal or near-normal levels of metanephrines and normetanephrines	Commonly extra-adrenal and primarily located in the head and neck region

### General principles in the surgical management of resectable PPGLs

Adrenalectomy remains the only curative treatment for locoregional disease despite advancements in systemic therapy [2]. To avoid a catecholamine crisis, which can lead to severe hypertension and other dangerous cardiovascular complications, it is important to maintain adequate control of blood pressure and heart rate before and during surgery. The recommended targets for blood pressure are 130/80 mmHg (1 mmHg = 0.133 kPa), while the heart rate should be between 60–80 beats per minute [2, 88]. Administering α-adrenergic receptor blockers for 10–14 days before surgery, adequate volume repletion, and a high salt diet significantly reduce postoperative mortality rates [89–92]. The objective of preoperative medical management is to control hypertension and tachycardia while also addressing volume depletion caused by catecholamines [91]. Alpha-blockers such as phenoxybenzamine, terazosin, and doxazosin are used to mitigate hypertension. The preferred choice is phenoxybenzamine, to be taken at a dosage of 10 mg twice a day or three times daily [93–95].

Prior to undergoing surgery, the administration of calcium-channel blockers and β-adrenergic receptor blockers may be considered in selected cases as a means of decreasing intraoperative hemodynamic instability and mitigating associated adverse outcomes [2, 96]. Clinicians must be aware that beta-adrenergic blockers should never be initiated first since an unopposed alpha-adrenergic receptor stimulation can further elevate blood pressure and can potentially cause a hypertensive crisis [97].

Robotic or Laparoscopic surgery are examples of minimally invasive techniques that may be utilised in lieu of an open procedure (anterior transabdominal, posterior, or flank approach). The primary goal during surgery is to avoid catecholamine surges. This is accomplished by minimizing mechanical pressure on the tumor during dissection, avoiding disruption of the adrenal capsule, and preventing the dissemination of tumor cells due to excessive manipulation [98, 99]. Patients with hereditary syndromes such as MEN2 or VHL may benefit from partial adrenalectomy to avoid steroid dependency instead of bilateral adrenalectomy [100]. PGLs are also treated primarily by surgical resection [101]. Preoperative medical preparation for functional PGLs is like that of PCCs, but surgical risks vary based on location [101].

Radiotherapy may be a preferable treatment option for large-sized jugular and jugulotympanic PGLs, involving the skull-base [102]. Adjuvant chemotherapy is not recommended after resection due to a lack of evidence from clinical trials [103]. Recent research has indicated that surgical debulking for metastatic PCCs can improve overall survival rates despite the possibility of low biochemical response rates [104–107]. This is because surgical debulking can help to reduce complications from further invasion, lower cardiovascular mortality, alleviate symptoms, and enhance the effectiveness of systemic treatment [84, 108].

### Surgical management of cluster 1 PPGLs

It is imperative to opt for surgery as the primary means of treating a locoregional disease, whenever possible [2]. When considering treatment options for cluster 1 PPGLs, it is recommended to prioritize total adrenalectomy over adrenal-sparing surgery. This is due to the high likelihood of recurrence and metastatic spread, particularly in tumors that have a mutation in the *SDHB* gene. It is important to carefully review each case and make individualized treatment decisions based on the specific circumstances and characteristics of the tumor [109].

Radiotherapy and systemic therapy may be options for PPGLs in the head and neck area depending on the patient’s age, life expectancy, comorbidities, performance status, and patients’ preferences [110]. Carotid body PGLs associated with *SDHB* and *SDHD* should be surgically excised if they reach a diameter of 1.5 cm or 2 cm, since larger tumor size is correlated with a greater probability of metastatic dissemination [3, 104, 110–113]. Neurological deficits, threat of brainstem compression, or refractory catecholamine secretions require surgical intervention as soon as possible [3]. For patients who exhibit advanced age, compromised performance status, poor prognosis, contraindications for surgical procedures, or simply a preference to avoid surgery, there exist several viable treatment modalities. External beam radiotherapy, stereotactic radiosurgery, proton beam radiation are feasible therapeutic options for individuals who exhibit advanced age, compromised performance status, poor prognosis, contraindications for surgical procedures, or simply a preference to avoid surgery [111, 112].

Following surgery, monitoring is suggested at periodic intervals every 3–12 months for the initial year, 6–12 months for the first three years, and thereafter annually for the duration of life in case of hereditary disease and for a minimum of 15 years in case of sporadic disease. Tailored follow-up regimens are recommended according to factors such as tumour size, location, and biochemical phenotype [2, 59]. The follow-up includes physical examination, careful monitoring for catecholamine-related symptoms and biochemical testing with plasma-free metanephrines or 24-h urine fractionated metanephrines [3, 111].

Those with inherited *SDHA/B* mutations, young age of onset (below 20 years), tumors beyond adrenals, and multiple tumors have high metastatic potential and thereby poor prognosis [103, 114–118]. Cluster 1A/B and 3 PPGLs have intermediate to high metastatic risk and hence MRI from skull base to pelvis is recommended every 12–24 months. Cluster 2 PPGLs need abdominal and pelvic MRI screenings every 5 years [95, 103].

### Surgical management of cluster 2 PPGLs and cluster 3 PPGLs

The surgical recommendations for cluster 2 and cluster 3 disease are identical to those for cluster 1. It is more common to recommend adrenal-sparing surgery for cluster 2-related local disease since it has a reduced incidence of recurrence and metastatic spread compared to cluster 1 disease [119, 120]. Cyclophosphamide, vincristine, and dacarbazine (CVD) chemotherapy is recommended for rapidly growing cluster 2 PPGLs, while radionuclide therapy, specifically ^131^I-MIBG or peptide receptor radionuclide therapy (PRRT), is recommended as the first-line therapy for slowly to moderately growing cluster 2 metastatic disease [2, 103]. This is discussed further in the systemic therapy. When there are no tumor-related symptoms and the tumor has a low metastatic potential, close monitoring may also be considered [89, 121].

### General principles in the management of non-resectable PPGLs

In order to alleviate symptoms associated with advanced disease and excessive catecholamine production by chromaffin cells, cytoreductive surgery is advised with or without adjuvant radiotherapy [89, 122, 123]. Radiotherapy can be considered for palliative treatment to provide symptomatic relief. Cryoablation and radiofrequency ablation are potential treatment options for the treatment of oligometastatic disease [124]. In the treatment of locally advanced and metastatic diseases, the current standard of care includes radionuclide therapy, chemotherapy, and tyrosine kinase inhibitors (TKIs).

### Overview of systemic therapy

Systemic therapy includes radionuclide therapy, chemotherapy, targeted therapy, and Immunotherapy. Radionuclide therapy is preferred for slow to moderate disease progression with low tumor burden, while chemotherapy is preferred for rapidly progressing disease with high tumor burden and when visceral crisis is present or imminent [2, 123, 125]. Typically, targeted therapy and immunotherapy are not the first option for treatment. Instead, they are often used to manage disease progression after chemotherapy or radionuclide therapy, or in clinical trials [2, 123, 125]. CVD chemotherapy, temozolomide [with or without poly(ADP-ribose) polymerase (PARP) or PARP inhibitors], PRRT, and hypoxia-inducible factor 2α (HIF-2a) inhibitors are all viable therapeutic alternatives for cluster 1-related PPGLs [2, 89, 103]. Aside from the fact that only 2% to 4% of metastatic PPGLs possess cluster 2 mutations, cluster 2-related diseases rarely require systemic therapy [126, 127]. Consequently, there is no treatment specific to cluster 2. Nevertheless, it is advisable to contemplate particular therapeutic alternatives for individuals in cluster 2. These alternatives comprise TKIs that selectively target kinase signaling pathways (e.g., lenvatinib, sunitinib, axitinib and cabozantinib), ^131^I-MIBG therapy and targeted inhibitors [MEK/ERK/RAF inhibitors and AKT/mammalian target of rapamycin complex 1 (mTORC1)/phosphoinositide 3-kinase (PI3K) inhibitors] [128–130].

## Radionuclide therapy

The three clusters are aligned in their approach to radionuclide therapy, utilizing ^131^I-MIBG radionuclide therapy in cases where a positive MIBG scan has been obtained, and employing PRRT in cases where a positive gallium DOTATAE scan has been obtained [2, 89, 103]. ^131^I-MIBG, including the novel high specific activity (HSA) ^131^I-MIBG, is the best-studied first-line therapeutic option for patients with PPGLs showing slow to moderate progression [2, 131–137].

Metastatic cluster 1 *SDHx*-related diseases, particularly those related to *SDHB,* may show lower positivity rates on ^123^I-MIBG scans [2, 138]. They are detected in the gallium DOTATAE scans and, therefore, treated with SSTR-based PRRT [139–147]. PRRT is recommended by the National Comprehensive Cancer Network (NCCN) and the European Society of Hypertension for treating PPGL [2]. Generally, patients tolerate PRRT well, and causes minimal side effects [2]. The need for treatment must be balanced with the possibility of severe bone marrow suppression, especially if radionuclide therapy is followed by chemotherapy [2]. TKIs could be an alternative second-line treatment to chemotherapy in patients who experience severe hematological toxicity as a result of their radionuclide therapy [2].

Nausea, myelosuppression, and fatigue are the most common side effects of radionuclide therapy [148]. Whenever a second application is indicated, a delay of at least 3 months should be considered until platelets and neutrophils are within normal ranges [149]. Potential long-term side effects include secondary hematological malignancies, such as myelodysplastic syndrome, acute myeloid leukemia, and acute lymphocytic leukemia [148].

## Chemotherapy

The Averbuch (CVD) scheme is the preferred chemotherapy treatment for rapidly spreading metastatic disease with a high tumor burden and for tumors unsuitable for radionuclide therapy [2, 88, 103]. The CVD regime (cyclophosphamide 750 mg/m^2^ on day 1, vincristine 1.4 mg/m^2^ on day 1, and dacarbazine 600 mg/m^2^ on days 1 and 2) is given at 21-day intervals [89]. This therapy is well-established and effective for aggressive and rapidly progressive PPGLs, especially in patients with *SDHB* mutations [108, 150, 151]. The most prevalent adverse events linked to the CVD regimen were myelosuppression, neuropathy, nausea, vomiting, and hemorrhagic cystitis. Less prevalent side effects are diarrhoea, alopecia, and oral ulcers [108].

As a form of maintenance therapy, it may be advisable to continue treatment with Temozolomide monotherapy if the patient has successfully completed 6–9 cycles of CVD chemotherapy or has been treated for a prolonged period with 20 cycles of CVD chemotherapy [150–152]. The standard Temozolomide regimen is 150 mg/m^2^ every 28 days on days 1–5 [89]. This is especially relevant for cases with *SDHB* mutations. In patients with *SDHB* mutation, who have limited performance status or co-morbidities and a lower disease burden, Temozolomide can be a substitute for CVD chemotherapy, achieving a disease control rate of up to 80% [progression-free survival (PFS) 13.3 months] and has more favourable side effect profile compared to CVD chemotherapy [3, 108, 151].

### TKIs

Angiogenesis is essential for the progression of metastatic PPGLs. TKIs inhibit intracellular signaling pathways involved in tumor development and target angiogenesis [153–156]. TKIs hinder the activation of vascular endothelial growth factor (VEGF) in PPGLs. Tumors with *SDHB* mutations have elevated levels of PDGF, Endothelin, angiopoietin, and VEGF. This makes TKIs effective in treating metastatic PPGLs that continue to progress after chemotherapy or radionuclide therapy [103]. However, the diminished effectiveness found in cluster 1-related malignancies may be due to its exclusive focus on receptor tyrosine kinase (RTK) signaling [103]. The ‘sunitinib in patients with progressive PGL or PCC (SNIPP)’ trial demonstrated that while sunitinib is beneficial for a significant number of patients with metastatic PPGLs, it yields a low objective response rate and an elevated prevalence of cumulative side effects and tumor resistance [156]. Cabozantinib, a TKI which preferentially targets vascular endothelial growth factor receptor 2 (VEGFR2) and cellular mesenchymal-epithelial transition (c-MET), is now being evaluated in clinical studies (NCT02302833) for the treatment of malignant PPGL [157]. More TKIs such as Pazopanib, Axitinib, and Lenvatinib, are currently under investigation [158, 159].

### Immunotherapy

The use of immunotherapy in cancer treatment is steadily growing, supplanting chemotherapy as the primary therapeutic option for several cancer types. Various phase I and II studies investigating the use of pembrolizumab as a single treatment showed limited effectiveness against tumors, independent of the presence of programmed death-ligand 1 (PD-L1) expression [160, 161]. Several more checkpoint inhibitors are now being assessed in clinical studies for advanced PPGLs. Now, two phase II studies (NCT03333616, NCT02834013) are looking at how well nivolumab and ipilimumab, which block the cytotoxic T-lymphocyte antigen 4 (CTLA-4), work together in metastatic PPGLs [162]. Further investigations are required to have a complete understanding of the function of immunotherapy in the treatment of PPGLs.

## Conclusions

The understanding about tumor metabolism in PPGLs and its clinical, diagnostic, prognostic and therapeutic implications have evolved significantly in the past few years. Discovery of new culprit genes and metabolic pathways are expected to improve the knowledge about the pathobiology of these uncommon tumors to enhance diagnostic and management strategies in future. Prompt recognition of the genetic abnormalities in patients with PPGLs through NGS would help clinicians to identify and categorize these tumors for appropriate design of management and follow up plan including genetic counseling of family members. More research involving multinational collaboration would empower clinicians to deal with this enigmatic disease in future.

## Abbreviations

^131^I-MIBG: ^131^I-metaiodobenzylguanidine
^18^F-FDOPA: 6-L-^18^F-fluorodihydroxyphenylalanine
3-MT: 3-methoxytyramine
^68^Ga-DOTATATE: ^68^Gallium-DOTA-Tyr3-octreotate
AM: adrenomedullin
CSDE1: cold shock domain-containing protein E1
CVD: cyclophosphamide, vincristine, and dacarbazine
D-2HG: D-2-hydroxyglutarate
DBH: dopamine β-hydroxylase
DDC: dihydroxyphenylalanine decarboxylase
DOPA: dihydroxyphenylalanine
EPAS1: endothelial Per-Arnt-Sim domain protein 1
FH: fumarate hydratase
FPGL: familial paraganglioma
HIFs: hypoxia-inducible factors
HNPGLs: head and neck paragangliomas
IDH: isocitrate dehydrogenase
LC: liquid chromatography
LC-MS/MS: liquid chromatography-tandem mass spectrometry
MAML3: mastermind like transcriptional coactivator 3
MAX: MYC associated factor X
MEN2: multiple endocrine neoplasia type 2
mRNA: messenger RNA
NF1: neurofibromatosis type 1
PCCs: pheochromocytomas
PET/CT: positron emission tomography/computed tomography
PGLs: paragangliomas
PNMT: phenylethanolamine N-methyltransferase
PPGLs: pheochromocytomas and paragangliomas
PRRT: peptide receptor radionuclide therapy
SDHx: succinate dehydrogenase complex
TH: tyrosine hydroxylase
TKIs: tyrosine kinase inhibitors
VEGF: vascular endothelial growth factor
VHL: von Hippel-Lindau

## Declarations

### Copyright

© The Author(s) 2024.
